# Plant-Derived Amino Acid-Based vs. Bovine-Derived Protein Human Milk Fortifiers in Preterm Infants at <34 Weeks’ Gestation: An Open-Label Pilot Randomized Controlled Trial

**DOI:** 10.3390/nu18152547

**Published:** 2026-08-04

**Authors:** Nikhil Kumar Mudmal, Viraraghavan Vadakkencherry Ramaswamy, Nasreen Banu Shaik, Laxman Basany, Abid Ali Hasan Ali, V. Sree Ramya

**Affiliations:** 1 Department of Neonatology, Ankura Hospital for Women and Children, Kukatpally, Hyderabad 500072, Telangana, India; mudmalnik@gmail.com (N.K.M.); banu_nimmi@yahoo.co.in (N.B.S.); drsreerv@gmail.com (V.S.R.); 2 Department of Neonatology, Fernandez Foundation, Hyderguda, Hyderabad 500029, Telangana, India; 3 Department of Pediatrics, Ankura Hospital for Women and Children, L. B. Nagar, Hyderabad 500074, Telangana, India; laxmanbasani@yahoo.co.in (L.B.);; 4 Department of Neonatology, Paramitha Women and Children’s Hospital, L. B. Nagar, Hyderabad 500074, Telangana, India; 5 Department of Pediatrics, Owaisi Hospital, Deccan College of Medical Sciences, Kanchan Bagh, Hyderabad 500058, Telangana, India

**Keywords:** preterm infant, enteral feed, human milk fortifier, amino acids, plant-based, bovine HMF, neonatal nutrition, randomized controlled trial

## Abstract

**Background:** Bovine milk protein-based human milk fortifiers (HMFs) are routinely used in preterm infants in low- and middle-income countries (LMICs) but are associated with potential adverse effects. Plant-derived amino acid-based HMFs are a promising alternative, but comparative data from randomized controlled trials (RCTs) are lacking. This pilot RCT aimed to evaluate the feasibility, safety, and growth outcomes of a novel plant-derived amino acid-based HMF compared to a bovine-derived whole-protein-based HMF in preterm infants at <34 weeks’ gestation. **Methods:** In this open-label, parallel pilot RCT, preterm infants at <34 weeks’ gestation were randomized to a plant-derived amino acid-based HMF (*n* = 66) or a bovine-derived whole-protein-based HMF (*n* = 70). Primary outcomes were time to reach 180 mL/kg/d enteral feeds and growth velocities to discharge and 40 weeks’ postmenstrual age (PMA). Feasibility and safety were also assessed. The analysis followed an intention-to-treat approach. The trial was registered (CTRI/2025/06/089133). **Results:** Baseline characteristics were comparable between groups. Times to reach enteral feeds of 180 mL/kg/d did not differ (adjusted Hazard Ratio 0.93, 95% CI 0.65, 1.33; *p* = 0.70). In-hospital weight gain was significantly higher in the plant-derived HMF group (adjusted mean difference (aMD) 3.20 g/kg/d, 95% CI 0.46, 5.95; *p* = 0.02). This benefit was most evident in subgroup analyses of infants with birth weight ≥1500 g (aMD 4.47 g/kg/d, 95% CI 0.09, 8.84; *p* = 0.04). Length, head circumference growth, and all safety outcomes were similar between groups. **Conclusions:** The plant-derived amino acid-based HMF demonstrated clinical feasibility and was well-tolerated. While it was associated with better in-hospital weight gain, differences in overall macronutrient density between the formulations may account for this finding. These results are hypothesis-generating and warrant future adequately powered trials.

## 1. Introduction

Optimal nutrition during the neonatal period is crucial for improving both short- and long-term outcomes in preterm infants, including survival, growth, and neurodevelopment [1,2,3,4]. Preterm neonates have markedly higher protein, energy and other micronutrient requirements when compared to term neonates [5,6]. Meeting these nutritional needs is limited by the immaturity of the gastrointestinal tract resulting in inadequate digestion, absorption, and assimilation. Deficiencies in protein and energy intake in the neonatal period in preterm infants are associated with poor outcomes such as extra-uterine growth restriction (EUGR), neurodevelopmental impairment, and metabolic syndrome in later life [3,4,5,6,7].

Mother’s own milk (MoM) is the most appropriate form of milk for very preterm (VPT) and very low birth weight (VLBW) infants due to its protective effects against mortality, necrotizing enterocolitis (NEC), late onset neonatal sepsis (LONS), and other morbidities [2,7]. When MoM is insufficient, pasteurized donor human milk (PDHM) is suggested as a reasonable alternative to preterm formula milk [8]. Since both MoM and PDHM do not meet the high protein, energy, and other nutritional requirements of preterm neonates, they are routinely supplemented with multi-nutrient human milk fortifiers (HMFs) [9,10,11]. A Cochrane review indicated that HMF use is associated with short-term beneficial effects on the anthropometric parameters of weight, length, and head-circumference, although evidence for reductions in major morbidities or improved long-term neurodevelopment is uncertain [1].

The protein constituent of routinely used HMFs in neonatal intensive care units (NICUs) is derived from bovine milk and is associated with several limitations [1,12]. Firstly, intact bovine proteins may be poorly digested by preterm neonates due to low intestinal proteolytic activity, and they have been shown to be associated with dysbiosis [13,14]. Secondly, exposure to bovine proteins in early neonatal life has been shown to be associated with intestinal inflammation and a higher risk of cow milk protein allergy in childhood [15,16]. Finally, the addition of bovine-derived HMF may result in increased osmolar load on the immature gut, perpetuating episodes of feed intolerance [17,18,19]. All of the above-mentioned factors could increase the risk of adverse effects such as mortality and NEC with the use of bovine-derived HMF [20,21].

In lieu of the aforementioned limitations of bovine-derived HMF, alternative fortifiers have been evaluated [22]. While one study has evaluated two different types of extensively hydrolyzed bovine protein fortifiers [23], others have studied human milk-derived fortifiers [21,24]. Several recently published meta-analyses have concluded that human milk-based fortifiers may significantly reduce the risk of mortality and NEC when compared to bovine-derived HMFs [22,25,26,27]. However, the beneficial effects of human milk-based fortifiers over bovine-derived HMF on other adverse outcomes such as growth, bronchopulmonary dysplasia (BPD) and retinopathy of prematurity (ROP) are inconclusive [28]. Further, the use of human milk-based fortifiers is limited by higher costs in low- and middle-income countries (LMICs) [29]. Protein supplementation in the form of free amino acids might be a potentially safer and efficacious alternative to bovine-derived whole or hydrolysed protein HMFs [30]. Amino acid-based HMFs are devoid of any bovine proteins and are derived from plant sources through microbial fermentation [31]. Since amino acids do not require luminal proteolysis, their absorption may be more efficient in preterm neonates [14,32].

Recently, a plant-derived amino acid-based multi-nutrient HMF was made available for use in preterm neonates. Small single-centre observational studies have reported it to be efficacious and safe with respect to growth and feed tolerance in preterm neonates [33,34]. An in vitro study found it to be associated with a lower osmolality compared to several bovine-based HMFs [17]. Despite these data, the feasibility, safety and comparative effectiveness of plant-derived amino acid HMF versus standard bovine-derived whole-protein HMFs have not been rigorously evaluated in a randomized controlled trial (RCT). Therefore, we conducted this open-label pilot RCT to compare a plant-derived amino acid HMF with a bovine-derived whole-protein HMF in preterm infants born at less than 34 weeks’ gestation. The primary aims were to assess feasibility and short-term growth outcomes (efficacy) and to evaluate safety by comparing the incidence of adverse events such as NEC, LONS, and mortality.

## 2. Materials and Methods

This prospective open-label parallel pilot RCT was conducted in two tertiary care NICUs in India from 19 June 2025 to 1 February 2026. The trial was approved by the Institutional Ethics Committee, and the protocol was registered in a trial registry (CTRI/2025/06/089133). Written informed consent was obtained from the parents or the caretakers of the neonates before enrollment. The reporting of this RCT adheres to the Consolidated Standards of Reporting Trials (CONSORT) statement for pilot RCTs [35].

### 2.1. Participants

*Inclusion criteria:* Preterm neonates born at less than 34 weeks’ gestation who were started on MoM or PDHM within 48 h of life.

*Exclusion criteria:* Neonates with gross congenital malformations and those whose caretakers refused consent.

### 2.2. Intervention (Plant-Derived Amino Acid-Based HMF)

The intervention group was administered a plant-derived amino acid-based HMF (HMF-Advance, Analeptik Biologicals LLP, Bengaluru, India). Each 1-g sachet delivers 4.5 kcal of energy and contains a free amino acid mixture comprising L-Arginine, L-Alanine, L-Cystine, L-Glutamine, L-Histidine, L-Isoleucine, L-Leucine, L-Lysine, L-Methionine, L-Proline, L-Serine, L-Threonine, L-Tryptophan, L-Valine, and Choline, collectively providing 0.35 g of protein equivalent, 0.24 g of carbohydrates, and 0.18 g of fats. Additionally, it includes essential fatty acids and long-chain polyunsaturated fatty acids: specifically, linolenic acid (2 mg), alpha-linolenic acid (100 mcg), docosahexaenoic acid (200 mcg), and arachidonic acid (200 mcg). Other pertinent constituents per gram include calcium (20 mg), phosphorus (10 mg), vitamin D (50 IU), vitamin A (100 IU), zinc (40 mcg), and other micronutrients.

### 2.3. Comparator (Bovine-Derived Whey Protein-Based HMF)

The control group was administered an HMF derived from bovine whey protein (Lactodex-HMF, Raptakos, Brett & Co. Ltd., Thane, India). Each 1-g sachet delivers 3.4 kcal of energy, comprising 0.27 g of complete protein sourced from whey protein concentrate and milk solids, 0.49 g of carbohydrates, and 0.04 g of fats. Additionally, the formulation includes calcium (16 mg), phosphorus (8 mg), vitamin D (133 IU), vitamin A (200 IU), zinc (40 mcg), iron (0.30 mg), and other essential micronutrients. Notably, this HMF does not include any free amino acids.

### 2.4. Enteral Feeding and Other Care Practices

Neonates born at ≥30 weeks of gestation were initiated on total enteral feeding at admission through an orogastric tube or cup depending upon their sickness profile. An enteral feed volume of 60 mL/kg/d in those with a birth weight of less than 1500 g and 80 mL/kg/d in others was provided on the first day of life. Neonates of <30 weeks’ gestation or those with antenatal doppler changes of absent or reversal of end-diastolic blood flow (A/REDF) in the umbilical artery were initiated on trophic feeds (12 mL/kg/d in neonates of <28 weeks’ gestation and 24 mL/kg/d in neonates born at 28^0/7^–29^6/7^ weeks’ gestation) immediately after admission through an orogastric tube. The volume of the enteral feeds was increased daily by 24 mL/kg/d in neonates of <28 weeks’ gestation and 36 mL/kg/d in others till a maximum of 200 mL/kg/d. Feeds were not administered ad libitum during the period of stay in the NICU as per the unit policy. Enteral nutrition was delivered through an orogastric tube at two-hour intervals, progressing to paladai (cup) feeding when the neonate attained 31–32 weeks’ PMA. The transition to direct breastfeeding commenced upon the establishment of suck-swallow-breathe coordination after 34 weeks’ PMA, ensuring a balance between direct breastfeeding and sufficient nutrient intake through the use of HMF. MoM was the preferred first choice of milk, and, if inadequate, PDHM was used to meet the target daily intake. Standard preterm formula was used when MoM was insufficient after discharge from the NICU. During the NICU stay, the nutrient intake and type of milk were strictly controlled; however, maintaining the same was not feasible after discharge in our setting. HMF was introduced when an enteral feed volume of 100 mL/kg/d was reached. The standardized fortification approach (1 g of HMF in 25 mL of milk) was used from the day of initiation of HMF. In accordance with the updated guidelines of the European Society of Pediatric Gastroenterology, Hepatology, and Nutrition, the nutrient intake for all neonates was calculated weekly to ensure the administration of maximal enteral nutrition doses in both groups. [36]. Fortification was continued until 40 weeks’ postmenstrual age (PMA). Pre-feed gastric aspiration was done only if the treating clinician or nurse had concerns on clinical examination. Single-strain probiotic supplementation (ProGG, *Lactobacillus rhamnosus* GG, 5 × 10^9^ CFU, Aristo Pharmaceuticals Pvt. Ltd., Mumbai, India) was provided from the time of initiation of enteral feeding at a dose of 2.5 × 10^9^ CFU/d until the neonate reached 60 mL/kg/d of enteral feeding and increased to 5 × 10^9^ CFU/d then on. Probiotics were continued until the neonate reached 34 weeks’ PMA. All other care practices were comparable between the two groups.

Those neonates who were born at less than 30 weeks’ gestation or those with antenatal doppler changes of A/REDF in the umbilical artery were initiated on TPN within 6 h of birth either through an umbilical venous catheter or a peripherally inserted central catheter line based on The American Society for Parenteral and Enteral Nutrition’s guidelines [37].

### 2.5. Outcomes

*Primary Outcomes*

- Time to reach enteral feed volume of 180 mL/kg/d.
- Weight gain (g/kg/d), head circumference increment (cm/week) and length increment (cm/week) from enrollment until discharge from the hospital and at 40 weeks’ PMA.

*Secondary Outcomes*

- Weight gain (g/kg/d) for different time intervals: birth until discharge, birth until 40 weeks’ PMA, from the day of regaining birth weight until 40 weeks’ PMA, and from the day of discharge until 40 weeks PMA (post hoc)
- Days to regain birth weight (post hoc)
- Incidence of feed intolerance (defined as necessity to keep the neonate nil by feeds for at least one day)
- NEC stage ≥ 2 (modified Bell’s staging) [38]
- All-cause mortality
- Late Onset Neonatal Sepsis (LONS) (Blood culture-proven sepsis or probable sepsis (clinical signs or symptoms indicative of sepsis with ≥2 altered biochemical sepsis parameters with the blood culture being negative))
- Requirement of red blood transfusion (post hoc)
- BPD (defined as oxygen requirement or any other form of respiratory support at 36 weeks’ PMA)
- ROP requiring intervention
- Metabolic bone disease
- Extra-uterine growth restriction (EUGR) defined as weight for age of less than the 10th centile at 40 weeks’ PMA
- Duration of hospital stay

Anthropometric measurements, including weight, length, and head circumference, were conducted by trained nursing personnel. These measurements utilized an electronic weighing scale, calibrated weekly with a precision of ±5 g, an infantometer, and a non-stretchable measuring tape, respectively.

### 2.6. Randomization Process, Allocation Concealment and Blinding

Neonates were randomized to the two groups in a 1:1 allocation ratio using stratified block randomisation. Randomisation sequence was generated using an online platform by an independent statistician not involved in the trial. Stratification was based on gestational age strata (<28 weeks, 28^0/7^–31^6/7^ weeks and 32^0/7^–33^6/7^ weeks). A block size of 4 was used within each gestational age category. Allocation concealment was ensured by using sequentially labelled opaque sealed envelopes. The treating clinician opened the envelope only if the neonate satisfied the inclusion criteria and informed consent was obtained. Blinding was not feasible because the HMF products differed in appearance and had to be added to each feed. Most infants were discharged before 40 weeks’ PMA, and providing parents with custom-made, masked sachets for home use was impractical and would have required manufacturer involvement, which was avoided to prevent conflicts of interest.

### 2.7. Sample Size

As this was a pilot RCT, it was not designed with sufficient power to detect clinically significant differences in growth or other efficacy outcomes. The primary objective was to evaluate feasibility and to generate preliminary data, including 95% confidence intervals (CIs), to inform sample size calculations for a future definitive trial. A sample size of 100 participants, with 50 in each group, was deemed appropriate for this purpose [39]. To account for a higher than expected lost to follow-up rate after discharge, we enrolled a total of 136 neonates (66 in the intervention group and 70 in the comparator group).

### 2.8. Data Collection and Analysis

Data were entered into pre-specified spreadsheets. Statistical analyses were performed using the R Software Version 4.3.2 (R Foundation for Statistical Computing, Vienna, Austria). Statistical significance was defined as a two-sided *p*-value < 0.05. The baseline characteristics of the enrolled neonates were compared between the two groups using appropriate statistical tests. While continuous variables are presented as medians with interquartile ranges (IQR) compared using the Mann–Whitney U test, categorical variables were analyzed using the Chi-square test or Fisher’s Exact test. To minimize bias associated with missing data and to adhere to the intention-to-treat (ITT) approach, missing values for primary and secondary outcomes were handled using multiple imputation by chained equations (MICE). Predictive mean matching (PMM) was used for continuous variables to ensure the imputed values remained within biologically plausible ranges. Fifty imputed datasets were generated. We used Rubin’s rules to generate valid standard errors and confidence intervals. We handled missing data based on the missing at random (MAR) assumption. We found MAR likely for several reasons. First, missing data due to death (5 infants, 3.7%) is not “missing” in the usual way, as we recorded outcomes at the time of death and included them in our analysis. Second, missing data from loss to follow-up after discharge (9 infants, 6.6%) was low and similar between groups. These losses were probably due to socioeconomic or logistic reasons common in LMICs (e.g., distance to hospital, family issues, returning to work). These factors are partly shown in our data (e.g., delivery method, mother’s health issues, multiple births). It is unlikely that loss to follow-up was directly caused by the infants’ anthropometric status at 40 weeks’ PMA (missing not at random), as families and healthcare providers involved in the trial probably did not know these outcomes at discharge. Growth velocities were analyzed using linear regression and are reported as mean differences (MD) with 95% CI. The primary outcome of time to reach an enteral feed volume of at least 180 mL/kg/day was compared using time-to-event analysis. Kaplan–Meier curves were generated to visualise the probability of reaching the target feed volume over time, and between-group differences were analyzed using the log-rank test. The Cox proportional hazards regression model was used to estimate the hazard ratio (HR) and 95% CI. To account for potential between-twins clustering, the Cox model utilized a cluster-robust variance estimator, with mother ID as the cluster variable. Multivariate adjusted analyses were performed for all the outcomes to account for the potential confounding factors of gestational age, birth weight, antenatal doppler abnormalities (AEDF or REDF in the umbilical artery) and the receipt of antenatal corticosteroids. Exploratory subgroup analyses were performed to evaluate treatment effects in different gestational age and birth weight strata (gestational age, <32 weeks vs. ≥32 weeks and birth weight, <1500 g vs. ≥1500 g) using multivariable linear regression models. Amongst the subgroups in which model convergence was not achieved due to a smaller number of neonates or data sparsity, adjustment was restricted to birth weight and gestational age alone. Per-protocol (PP) analyses for all the primary and secondary growth outcomes were performed to evaluate the robustness of the primary ITT analyses.

## 3. Results

**Figure 1:**
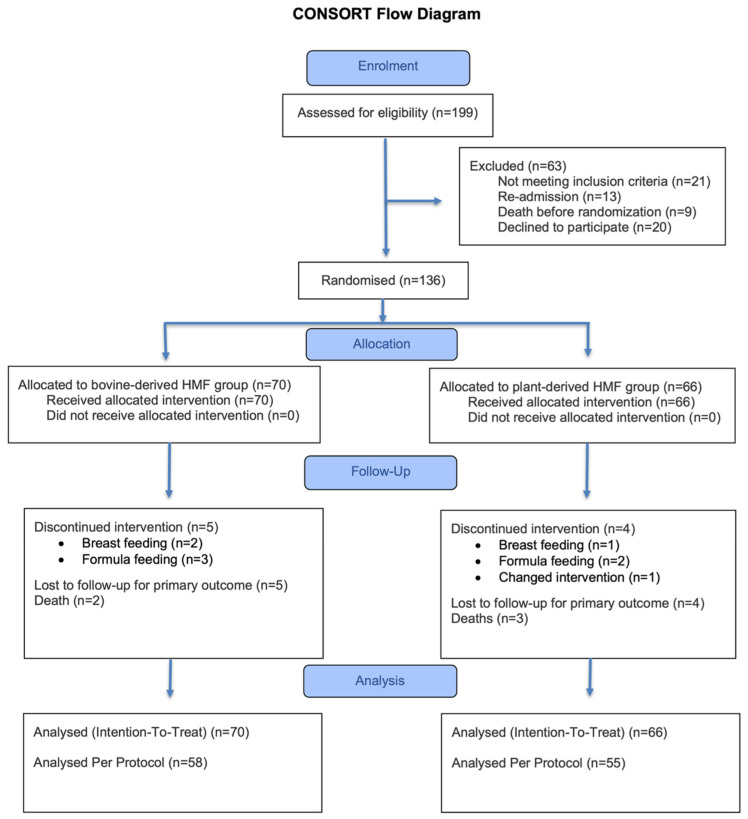
CONSORT flow diagram.

A total of 199 preterm neonates were assessed for eligibility from 19 June 2025 to 1 January 2026. Among them, 136 neonates met the inclusion criteria and were randomized; 66 neonates to the plant-derived amino acid HMF group and 70 neonates to the bovine-derived protein HMF group. Of the 136 neonates enrolled in the trial, all reached the primary outcome of time to reach enteral feed volume of 180 mL/kg/d. A total of 11 neonates in the plant-derived HMF group and 12 neonates in the bovine-derived HMF group either discontinued the intervention, were lost to follow-up or died. While the outcomes of all the neonates randomized to the two groups were analyzed in the primary ITT analyses, PP sensitivity analyses for the primary and secondary growth outcomes included 113 neonates (plant-derived HMF, *n* = 55; bovine-derived HMF group, *n* = 58) who fully adhered to the study protocol and completed the study follow-up until 40 weeks’ PMA. Figure 1 illustrates the participant flow of the trial. The baseline demographic and clinical variables were similar between the two groups, provided in Table 1.

### 3.1. Primary Outcomes

#### 3.1.1. Time to Reach Enteral Feed Volume of 180 mL/kg/d

**Figure 2:**
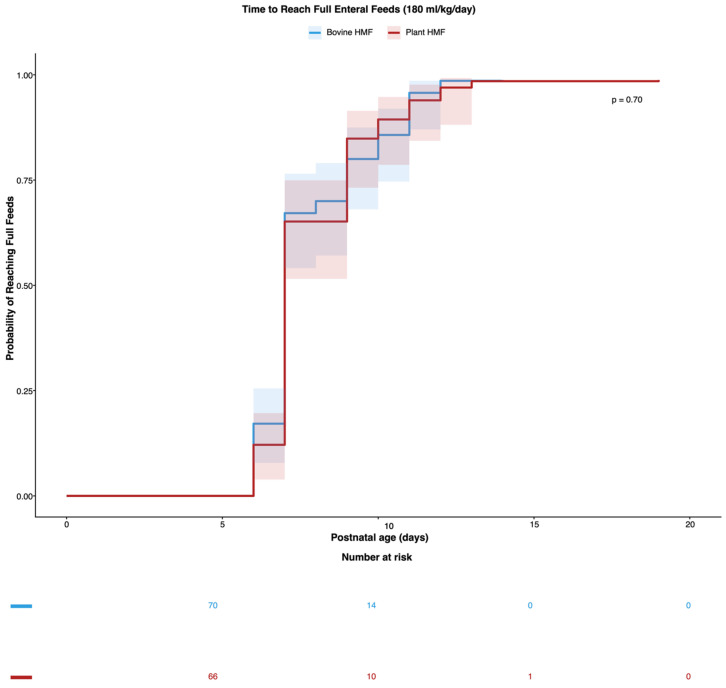
Intention-to-treat analysis for time to reach enteral feed volume of 180 mL/kg/d (Kaplan–Meier survival estimates) between plant-derived amino acid-based HMF and bovine-derived protein-based HMF groups.

Neonates in both groups had a similar rate of progression of enteral feeding with no significant difference in the time to reach an enteral feed volume of 180 mL/kg/d (log-rank test, *p* = 0.70; HR (95% CI), 0.95 (0.69, 1.33), *p* = 0.78)). The Kaplan–Meier survival curves are provided in Figure 2. After accounting for the potential confounding factors of the receipt of antenatal corticosteroids, umbilical artery doppler abnormalities, gestational age and birth weight, the adjusted multivariate Cox proportional hazards regression analysis also indicated no significant differences between the two groups (aHR (95% CI), 0.93 (0.65, 1.33); *p* = 0.70)).

#### 3.1.2. Anthropometric Parameters from Enrollment Until Discharge and 40 Weeks’ PMA

**Figure 3:**
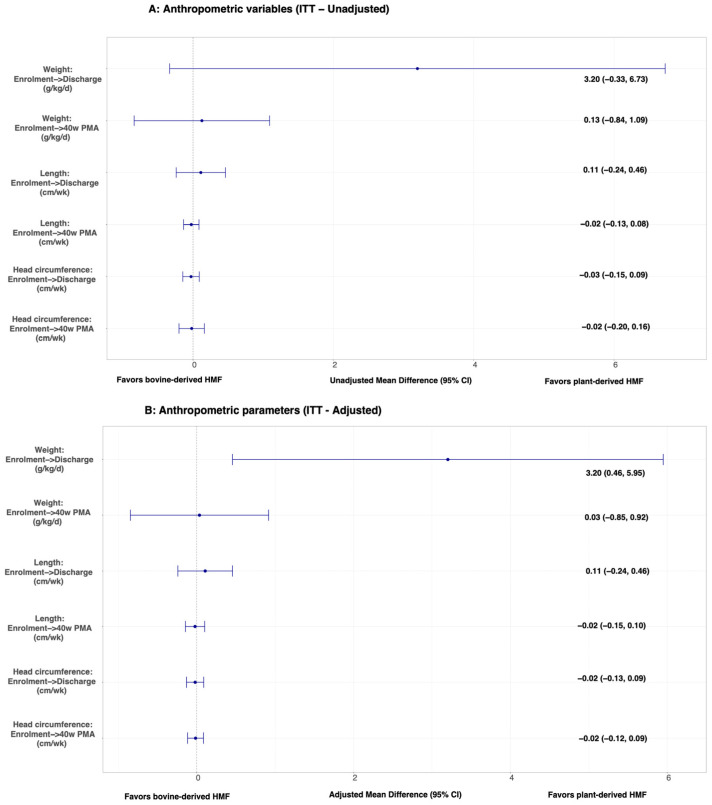
Unadjusted and adjusted intention-to-treat analyses of growth velocities. (weight, length, and head circumference) between plant-derived amino acid-based HMF and bovine-derived protein-based HMF groups.

The weight gain velocity, increments in length and head circumference from enrollment until discharge and 40 weeks’ PMA—were similar between the two groups. (Table 2 and Figure 3). Multivariable linear regression adjusting for the aforementioned effect modifiers indicated that the weight gain velocity from enrollment until discharge was significantly higher in plant-derived amino acid HMF group when compared to the bovine-derived HMF group (aMD (95% CI), 3.20 g/kg/d (0.46, 5.95); *p* = 0.02)). All other growth variables were similar between the two groups. (Table 2 and Figure 3).

### 3.2. Secondary Outcomes

Weight gain velocity was assessed across different epochs: namely, from birth until discharge and 40 weeks’ PMA, from the day of regaining birth weight until 40 weeks’ PMA, and from hospital discharge until 40 weeks’ PMA. Since some neonates were discharged before regaining birth weight, we could not analyse the weight velocity from the day of regaining birth weight until hospital discharge. The aMD in the rate of weight gain for all the different epochs was similar between the two groups (Table 3 and Supplemental Figure S1). The other secondary outcomes of days to regain birth weight, number of feed intolerance days, duration of hospital stay, and safety outcomes of mortality and other morbidities were also not significantly different between the two groups (Table 3 and Supplemental Figure S2). Initially, the proportion of neonates classified as Small for Gestational Age (SGA; weight below the 10th percentile) was 7.1% in the bovine group compared to 12.1% in the plant group (*p* = 0.49). By 40 weeks’ postmenstrual age (PMA), the incidence of EUGR (weight below the 10th percentile) did not differ significantly between the two cohorts, with rates of 14.0% and 18.3%, respectively (*p* = 0.63). The Kaplan–Meier survival curves for days to regain birth weight is provided in Supplemental Figure S3.

#### 3.2.1. Sub-Group Analyses (Post Hoc)

Sub-group analyses were performed based on gestational age (<32 weeks vs. ≥32 weeks) and birth weight (<1500 g vs. ≥1500 g) for the primary growth outcomes after adjusting for the confounding variables. For the outcome of rate of weight gain from enrollment until discharge, the primary ITT analysis had indicated beneficial effects of plant-derived amino acid-based HMF amongst all the enrolled neonates (aMD (95%CI), 3.20 g/kg/d (0.46, 5.95); *p* = 0.02)). Subgroup analyses revealed that this effect was more pronounced in larger infants with birth weight ≥1500 g (aMD (95%CI), 4.47 g/kg/d (0.09, 8.84); *p* = 0.04)). No significant subgroup differences were observed between the two groups for the outcomes of length and head circumference increments (Supplemental Table S1 and Supplemental Figure S4).

#### 3.2.2. Sensitivity Analyses

PP analyses were performed for the primary outcomes of weight gain, length increment and head circumference increments from enrollment until discharge. The unadjusted PP analyses showed similar results with no statistically significant differences between the two groups. Similar to the adjusted ITT analysis, the adjusted PP analysis also showed a statistically significant difference in the weight velocity from enrollment until discharge that favoured the plant-derived amino acid-based HMF group (aMD (95%CI), 3.96 g/kg/d (1.44, 6.47); *p* = 0.002)). The robustness of the primary analyses was further established with similar results observed for the outcomes of length and head circumference increments from enrollment until discharge and at 40 weeks’ PMA, and secondary weight outcomes across different time periods (Supplemental Table S2 and Supplemental Figure S5). PP subgroup analyses showed favourable effects of plant-derived amino acid-based HMF for the outcome of rate of weight gain from enrollment until discharge in neonates with birth weight ≥ 1500 g (aMD (95%CI), 5.40 g/kg/d (1.54, 9.26); *p* = 0.006)), as seen in the ITT analyses. Additionally, a similar effect favouring plant-derived amino acid-based HMF was also seen in neonates born at ≥32 weeks’ gestation (aMD (95%CI), 7.47 g/kg/d (1.63, 13.31); *p* = 0.01)) (Supplemental Table S3 and Supplemental Figure S6).

The absolute weight, length, and head circumference from birth through 40 weeks PMA were similar between the two groups for ITT and PP analyses (Supplemental Figure S7). The MD in weight, length and head circumference was neither clinically nor statistically significant between the two groups at discharge and at 40 weeks’ PMA.

## 4. Discussion

In this pilot RCT, we assessed the feasibility, safety, and preliminary efficacy regarding the growth parameters of a novel plant-derived amino acid-based HMF in comparison to the conventionally used bovine-derived whole-protein HMF in preterm neonates born at less than 34 weeks of gestation. The results of this RCT, including the primary outcomes, are exploratory and require validation in future definitive trials.

The efficacy of the plant-derived amino acid-based HMF seen in our exploratory trial could be attributed to a multitude of biologically plausible reasons. The primary mechanism likely involves the digestive physiology of the preterm gut. Demers-Mathieu et al. reported that preterm neonates have significantly lower intestinal proteolytic activity compared to term infants [14]. By providing nitrogen in its elemental amino acid form, our intervention likely bypassed the rate-limiting hydrolysis required for bovine protein, thereby enhancing nitrogen retention more effectively. Also, the avoidance of exposure to bovine antigens in the plant-derived HMF group might have maintained a balanced oxidant vs. antioxidant profile and promoted a favourable microbiome milieu in the intestinal tract [40,41]. It is noteworthy that the plant-derived HMF group had a higher proportion of neonates with antenatal doppler abnormalities compared to the group who received bovine-derived HMF, although this difference did not reach statistical significance. Given that such infants are recognized to be at an elevated risk for morbidities including EUGR, NEC, and feed intolerance, we accounted for this potential confounding factor in all multivariable regression analyses. The observation that in-hospital weight gain remained significantly greater in the plant-derived HMF group even after adjustment enhances our confidence in this exploratory finding.

It is crucial to emphasize that this trial compared two distinct formulations, not just protein sources. An important interpretive consideration is that the observed difference in weight gain may be partially attributed to the higher energy density of the plant-derived HMF (4.5 kcal/g compared to 3.4 kcal/g) and its greater protein equivalent (0.35 g/g versus 0.27 g/g). With the standardized fortification strategy employed in this RCT, the plant HMF group likely received additional calories and proteins compared to the bovine HMF group. This cumulative nutrient surplus could have independently contributed to the observed weight gain advantage. Moreover, head circumference, a crucial indicator of brain growth, did not differ significantly between the groups (*p* = 0.72), suggesting that the weight gain advantage may be due to non-neurological tissue accretion. Future research should aim to distinguish the effects of protein sources from those of overall energy density. Moreover, the nutritional profile of amino acid-based HMF, characterized by a higher content of essential fatty acids and a lower carbohydrate content, may have also contributed to improved in-hospital weight gain [42,43]. Furthermore, an in vitro study evaluating various HMFs, including those used in our trial, revealed through direct comparison that the amino acid-based fortifier yielded a lower reconstituted osmolality compared to the bovine-based protein HMF [17]. This is probably significant, as a higher osmolar load has been shown to be associated with altered intestinal mucosal integrity in pre-clinical studies, which could adversely affect nutrient absorption [19,44,45]. We acknowledge that the higher protein content in the plant-derived amino acid-based HMF in conjunction with the use of the standardized fortification approach would have possibly contributed to the observed better in-hospital weight gain.

The observed in-hospital weight gain benefit with plant-derived amino acid-based HMF was primarily evident in more mature preterm infants, possibly due to their enhanced metabolic capacity to utilize higher protein intake [46]. These findings should be interpreted as hypothesis-generating, as this was a post hoc exploratory sub-group analysis. This subgroup is critically relevant in the context of neonatal care in LMICs, as it represents a large proportion of preterm admissions in LMICs who are often excluded in HMF trials [47]. However, this growth advantage did not extend until 40 weeks’ PMA. While the period from discharge until 40 weeks PMA was uncontrolled, this transient effect is consistent with that reported in the literature, where the post-discharge cessation of fortification due to non-compliance or other reasons such as transitioning to exclusive breast feeding typically leads to convergence in growth trajectories [11,48]. Moreover, the in-hospital weight gain benefit did not result in significant differences in absolute weight, length, or head circumference at discharge or at 40 weeks’ PMA between the two groups.

An exclusive human milk diet supplemented with a fortifier derived from human milk presents a promising alternative to bovine-derived HMF. However, its feasibility, cost, and ethical considerations in LMICs remain the subjects of ongoing debate [49]. Furthermore, a recent Bayesian meta-analysis has challenged the perceived superiority of human milk-derived fortifiers over their bovine-derived counterparts [28]. In this context, plant-derived amino acid HMFs may offer a more scalable and practical alternative for use in LMICs. Additionally, alternative fortifiers, such as those based on extensively hydrolyzed bovine protein, have been assessed [23]. Although these extensively hydrolyzed bovine protein HMFs exhibit reduced antigenicity compared to intact whey, they are not available in our region.

This RCT has several limitations. This was a pilot RCT involving two centres, and hence it is underpowered for critical outcomes. While the trial successfully achieved the required sample size, the loss to follow-up after discharge, although not considered significant, may have introduced bias due to missing data [50]. The open-label design is a significant limitation. We recognize that the absence of blinding of the interventions to healthcare providers or caretakers may have introduced potential performance and detection biases, particularly concerning subjective outcomes such as feed intolerance. Our results are not generalizable to extremely low gestational age neonates, as they constituted only a minor proportion of those enrolled in our trial. We could not compare the nutrient intake accurately in some neonates after discharge from the hospital because these neonates were transitioned to direct breast feeding with or without variable amounts of formula feeds. While we assessed various pre-specified ‘critical’ and ‘important’ outcomes as directed by the COMET (Core Outcome Measures in Effectiveness Trials) initiative [51,52], we recognize that more direct metabolic endpoints, such as plasma amino acid profiles, were not evaluated primarily due to resource limitations. Plasma amino acid profiles would offer crucial insights into the potential causal pathway leading to clinically significant outcomes, such as growth. These are key points that should be focused on in future RCTs.

A notable limitation of this study is the assessment of weight gain solely in terms of total mass without analyzing the specific composition of this gain, particularly the distinction between lean (fat-free) mass and fat mass. Infant body composition, which is significantly influenced by variations in protein and energy intake, is a more precise indicator of nutritional quality and a superior predictor of long-term neurodevelopmental and cardiometabolic outcomes compared to weight alone [53]. Therefore, without understanding the tissue composition, the observed advantage in weight gain cannot be conclusively considered beneficial. Finally, as a pilot study, the sample size was notably inadequate to rule out significant harm or to identify differences in rare but critical safety outcomes, such as NEC and mortality. Consequently, the safety data presented here are preliminary and do not indicate safety equivalence.

There were several strengths as well. To the best of our knowledge, this is the only RCT that has evaluated a plant-derived amino acid-based HMF vs. bovine-derived protein-based HMF. Further, the statistical analyses were comprehensive and robust, accounting for lost to follow-up using recommended approaches with adherence to the CONSORT for pilot RCTs guidance. Finally, this trial addressed a significant knowledge gap concerning the use of HMF in relatively mature preterm neonates, specifically those with a gestational age of ≥32 weeks from LMICs, who were monitored beyond hospital discharge. Although the findings of our pilot trial may not be applicable to extremely preterm neonates, future comprehensive trials from LMICs are expected to include a similar participant demographic. The preliminary effect estimates derived from our trial could substantially inform the design and implementation of such trials in LMICs.

## 5. Conclusions

In conclusion, this pilot RCT demonstrates the feasibility and tolerability of a novel plant-derived amino acid-based HMF when compared to bovine-derived protein-based HMF. The observed in-hospital weight gain benefit with plant-derived amino acid-based HMF is hypothesis-generating and may reflect its higher macronutrient density. These preliminary findings inform the design of future multi-center RCTs adequately powered to assess critical safety outcomes.

## Figures and Tables

**Table 1:** Baseline demographic and clinical variables between bovine-derived protein-based HMF and plant-derived amino acid-based HMF groups.

Variable	Bovine HMF (*n* = 70)	Plant HMF (*n* = 66)	*p*-Value
**Gestational age (w)**	31.0 [29.0, 32.0]	31.0 [30.0, 33.0]	0.52
**Birth weight (g)**	1505 [1192, 1769]	1565 [1200, 1751]	0.75
**Admission temperature (°C)**	36.5 [36.2, 36.6]	36.5 [36.4, 36.7]	0.43
**Age at enrollment (h)**	48 [48, 96]	48 [48, 96]	0.52
**Weight at enrollment (g)**	1472 [1142, 1745]	1515 [1170, 1720]	0.73
**Length at enrollment (cm)**	40.0 [38.0, 42.0]	41.0 [38.1, 43.0]	0.55
**Head circumference at enrollment (cm)**	29.0 [27.5, 30.0]	29.0 [28.0, 30.0]	0.55
**Gestational age category**			0.41
<28 w	7 (10.0%)	3 (4.5%)	-
28–< 32 w	32 (45.7%)	35 (53.0%)	-
≥32 w	31 (44.3%)	28 (42.4%)	-
**Birth weight category**			0.46
<1000 g	10 (14.3%)	5 (7.6%)	-
1000–1499 g	23 (32.9%)	24 (36.4%)	-
≥1500 g	37 (52.9%)	37 (56.1%)	-
**Weight for age**			0.49
AGA or LGA	65 (92.9%)	58 (87.9%)	-
SGA	5 (7.1%)	8 (12.1%)	-
**Multiple gestation**	29 (41.4%)	22 (33.3%)	0.42
**Gender**			0.93
Male	43 (61.4%)	40 (60.6%)	-
Female	27 (38.6%)	26 (39.4%)	-
**Maternal hypertension**	14 (20.0%)	17 (25.8%)	0.55
**Maternal diabetes**	8 (11.4%)	7 (10.6%)	1.00
**Maternal fever**	8 (11.4%)	7 (10.6%)	1.00
**Umbilical artery doppler changes (A/REDF)**	3 (4.3%)	9 (13.6%)	0.10
**Antenatal steroids**			0.49
Complete	33 (47.1%)	34 (51.5%)	-
Incomplete	22 (31.4%)	23 (34.8%)	-
Not Received	15 (21.4%)	9 (13.6%)	-
**Deferred cord clamping ≥ 60 s**	33 (47.1%)	32 (48.5%)	1.00
**Mode of delivery**			0.26
LSCS	58 (82.9%)	60 (90.9%)	-
VD	12 (17.1%)	6 (9.1%)	-
**Any resuscitation required**	9 (12.9%)	8 (12.1%)	1.00
**Total parenteral nutrition requirement on day 1**	16 (22.9%)	14 (21.2%)	0.98
**Time of initiation of enteral nutrition (h)**	2.0 [2.0, 2.0]	2.0 [1.0, 2.0]	0.26
**MoM on day 7 (%)**	100 [65, 100]	100 [80, 100]	0.35

**Abbreviations:** AGA, appropriate for gestational age; A/REDF, absent or reversal of flow in the umbilical artery; °C, degree Celsius; cm, centimetres; g, grams; h, hours; LGA, large for gestational age; LSCS, lower segment caesarean section; MoM, mother’s own milk; s, seconds; SGA, small for gestational age; VD, vaginal delivery; w, weeks.

**Table 2:** Unadjusted and adjusted intention-to-treat analyses of growth velocities (weight, length, and head circumference) between plant-derived amino acid-based HMF and bovine-derived protein-based HMF groups.

Anthropometric Parameter	Bovine HMF (*n* = 70) Imputed Mean (SD) *^+^	Plant HMF (*n* = 66) Imputed Mean (SD)	MD (95% CI) *	*p*-Value	aMD (95% CI) *^+^	*p*-Value
**Weight ^++^**						
Enrollment until discharge	13.3 (6.8)	16.5 (7.2)	3.20 (−0.33, 6.73)	0.08	3.20 (0.46, 5.95)	0.02
Enrollment until 40 w PMA	10.6 (3.2)	10.7 (3.6)	0.13 (−0.84, 1.09)	0.80	0.03 (−0.85, 0.92)	0.95
**Length ^++^**						
Enrollment until discharge	0.95 (0.45)	1.06 (0.52)	0.11 (−0.24, 0.46)	0.53	0.11 (−0.24, 0.46)	0.54
Enrollment until 40 w PMA	0.65 (0.28)	0.63 (0.30)	−0.02 (−0.13, 0.08)	0.65	−0.02 (−0.15, 0.10)	0.75
**HC ^++^**						
Enrollment until discharge	0.65 (0.22)	0.62 (0.24)	−0.03 (−0.15, 0.09)	0.63	−0.02 (−0.13, 0.09)	0.72
Enrollment until 40 w PMA	0.52 (0.18)	0.50 (0.19)	−0.02 (−0.20, 0.16)	0.83	−0.02 (−0.12, 0.09)	0.71

**Abbreviations:** aMD, adjusted mean difference; CI, confidence interval; HC, head circumference; HMF, human milk fortifier; MD, mean difference; * intention-to-treat analyses; ^+^ Multivariable linear regression analyses, adjusted for gestational age, birth weight, receipt of antenatal corticosteroids, antenatal doppler abnormalities. Group-specific descriptives denote pooled estimates derived across 50 datasets generated by multiple imputation using chained equations (MICE) under the intention-to-treat principle to handle missing data due to longitudinal dropouts, structural alignment, or early discharge; ^++^ Weight velocity in g/kg/d, length and HC increments in cm/w.

**Table 3:** Adjusted intention-to-treat analyses of secondary and safety outcomes between plant-derived amino acid-based HMF and bovine-derived protein-based HMF groups.

Outcome Variables	Bovine HMF (*n* = 70) *^+^	Plant HMF (*n* = 66) *^+^	aMD/aRR *^+^ (95% CI) (Plant HMF vs. Bovine HMF)	aRD (95% CI) *^+^ (Plant HMF vs. Bovine HMF)	*p*-Value
**Rate of Weight Gain (g/kg/d)**					
Birth until discharge	1.59 [−7.10, 8.29]	2.49 [−1.00, 8.17]	3.15 (−0.17, 6.48)	NA	0.06
Birth until 40 w PMA	10.49 [8.91, 12.09]	10.32 [8.88, 11.74]	0.05 (−0.80, 0.90)	NA	0.91
Day of regaining BW until 40 w PMA	13.12 [11.39, 14.58]	12.70 [11.21, 14.97]	−0.24 (−1.58, 1.09)	NA	0.72
Discharge until 40 w PMA	12.58 [11.04, 15.04]	12.25 [10.78, 14.71]	−0.08 (−1.18, 1.02)	NA	0.89
**Days to regain BW**	12.00 [10.00, 14.00]	13.08 [9.00, 15.98]	1.40 (−1.30, 4.09)	NA	0.31
**No. of feed intolerance days**	0.00 [0.00, 0.01]	0.00 [0.00, 0.01]	0.22 (−0.31, 0.75)	NA	0.41
**Duration of hospital stay (d)**	14.00 [7.00, 28.00]	14.00 [9.00, 24.00]	−2.61 (−9.11, 3.89)	NA	0.43
**Receipt of RBC transfusion**	11/67 (16.4%)	10/62 (16.1%)	1.01 (0.43, 2.36)	0.17 (−12.61, 12.94)	0.98
**NEC (≥Stage 2)**	2/66 (3.0%)	3/61 (4.9%)	1.56 (0.26, 9.56)	1.63 (−4.97, 8.24)	0.63
**LONS**	21/66 (31.8%)	19/62 (30.6%)	0.96 (0.52, 1.78)	−1.33 (−17.41, 14.76)	0.89
**BPD**	5/67 (7.5%)	7/62 (11.3%)	1.48 (0.47, 4.71)	3.50 (−6.34, 13.34)	0.50
**ROP requiring intervention**	13/67 (19.4%)	8/62 (12.9%)	0.69 (0.29, 1.66)	−5.87 (−18.64, 6.90)	0.41
**MBD**	12/67 (17.9%)	14/62 (22.6%)	1.26 (0.58, 2.74)	4.64 (−9.12, 18.40)	0.55
**EUGR at 40 w PMA**	9/64 (14.1%)	11/60 (18.3%)	1.24 (0.52, 3.00)	3.32 (−9.23, 15.88)	0.63
**Mortality**	2/70 (2.9%)	3/65 (4.6%)	1.59 (0.27, 9.22)	1.69 (−4.67, 8.05)	0.67
**Mortality or LTFU**	12/70 (17.1%)	11/66 (16.7%)	0.97 (0.46, 2.05)	−0.48 (−13.08, 12.13)	1.00

**Abbreviations:** BPD, bronchopulmonary dysplasia; CI, confidence interval; EUGR: extra-uterine growth restriction; HMF, human milk fortifier; interquartile range; LONS, late onset neonatal sepsis; LTFU, lost to follow-up; MBD, metabolic bone disease; aMD, adjusted mean difference; NA, not applicable; NEC, necrotizing enterocolitis; PMA: post-menstrual age; RBC, red blood cell; aRD, adjusted risk difference; aRR, adjusted risk ratio; ROP, retinopathy of prematurity. * Intention-to-treat analyses. For categorical variables, observed event counts (*n*/N) are derived strictly from complete-case data. Effect estimates (aRR and aRD) are pooled via Rubin’s rules across 50 datasets generated by multiple imputation using chained equations (MICE) to account for missing covariates, which accounts for slight divergence from raw proportional counts. ^+^ Multivariable linear regression (continuous variables) and modified Poisson regression with robust error variance (categorical variables) analyses, adjusted for gestational age, birth weight, receipt of antenatal corticosteroids, antenatal doppler abnormalities.

## Data Availability

Data related to this trial could be provided on request to the corresponding author.

## Supplementary Materials

The following supporting information can be downloaded at: https://www.mdpi.com/article/10.3390/nu18152547/s1, Figure S1: Adjusted Intention-to-Treat Analyses Comparing Weight Gain Velocity Over Time Between Plant-Derived and Bovine-Derived HMF Groups; Figure S2: Adjusted Intention-to-Treat Analyses of Secondary and Safety Outcomes Between Plant-Derived and Bovine-Derived HMF Groups; Figure S3: Adjusted Intention-to-Treat Analysis for Time to Regain Birth Weight (Kaplan-Meier Survival Estimates) Between Plant-Derived and Bovine-Derived HMF Groups; Figure S4: Adjusted Intention-to-Treat Subgroup Analyses of Growth Velocities (Weight, Length, and Head Circumference) Between Plant-Derived and Bovine-Derived HMF Groups; Figure S5: Adjusted and Unadjusted Per-Protocol Analyses of Growth Velocities (Weight, Length, and Head Circumference) Between Plant-Derived and Bovine-Derived HMF Groups; Figure S6: Adjusted Per-Protocol Subgroup Analyses of Growth Velocities (Weight, Length, and Head Circumference) Between Plant-Derived and Bovine-Derived HMF Groups; Figure S7: Unadjusted Longitudinal Growth Trajectories Between Plant-Derived vs. Bovine-Derived HMF groups; Table S1: Adjusted Intention-to-Treat Subgroup Analyses of Growth Velocities (Weight, Length, and Head Circumference) Between Plant-Derived and Bovine-Derived HMF Groups; Table S2: Adjusted and Unadjusted Per-Protocol Analyses of Growth Velocities (Weight, Length, and Head Circumference) and Weight Gain Velocity Over Time Between Plant-Derived and Bovine-Derived HMF Groups; Table S3: Adjusted Per-Protocol Subgroup Analyses of Growth Velocities (Weight, Length, and Head Circumference) and Weight Gain Velocity Over Time Between Plant-Derived and Bovine-Derived HMF Groups.
