# Supplementary material for: Plant-Derived Amino Acid-Based vs. Bovine-Derived Protein Human Milk Fortifiers in Preterm Infants at <34 Weeks’ Gestation: An Open-Label Pilot Randomized Controlled Trial
Source: Nutrients. 2026 Aug 4;18(15):2547. doi: 10.3390/nu18152547 (PMC13467811; doi:10.3390/nu18152547)
Supplement: Supplementary file 1 [file nutrients-18-02547-s001.zip › nutrients-4265863-supplementary.pdf]

Supplement File

Supplement Figure S1: Adjusted Intention-to-Treat Analyses Comparing Weight Gain Velocity Over Time Between Plant-Derived and Bovine-Derived HMF Groups

Supplement Figure S2: Adjusted Intention-to-Treat Analyses of Secondary and Safety Outcomes Between Plant-Derived and Bovine-Derived HMF Groups

Supplement Figure S3: Adjusted Intention-to-Treat Analysis for Time to Regain Birth Weight (Kaplan-Meier Survival Estimates) Between Plant-Derived and Bovine-Derived HMF Groups

Supplement Figure S4: Adjusted Intention-to-Treat Subgroup Analyses of Growth Velocities (Weight, Length, and Head Circumference) Between Plant-Derived and Bovine-Derived HMF Groups

Supplement Figure S5: Adjusted and Unadjusted Per-Protocol Analyses of Growth Velocities (Weight, Length, and Head Circumference) Between Plant-Derived and Bovine-Derived HMF Groups

Supplement Figure S6: Adjusted Per-Protocol Subgroup Analyses of Growth Velocities (Weight, Length, and Head Circumference) Between Plant-Derived and Bovine-Derived HMF Groups

Supplement Figure S7: Unadjusted Longitudinal Growth Trajectories Between Plant-Derived vs. Bovine-Derived HMF groups.

Supplement Table S1: Adjusted Intention-to-Treat Subgroup Analyses of Growth Velocities (Weight, Length, and Head Circumference) Between Plant-Derived and Bovine-Derived HMF Groups

Supplement Table S2: Adjusted and Unadjusted Per-Protocol Analyses of Growth Velocities (Weight, Length, and Head Circumference) and Weight Gain Velocity Over Time Between Plant-Derived and Bovine-Derived HMF Groups

Supplement Table S3: Adjusted Per-Protocol Subgroup Analyses of Growth Velocities (Weight, Length, and Head Circumference) and Weight Gain Velocity Over Time Between Plant-Derived and Bovine-Derived HMF Groups

Supplement Figure S1: Adjusted Intention-to-Treat Analyses Comparing Weight Gain Velocity Over Time Between Plant-Derived and Bovine-Derived HMF Groups

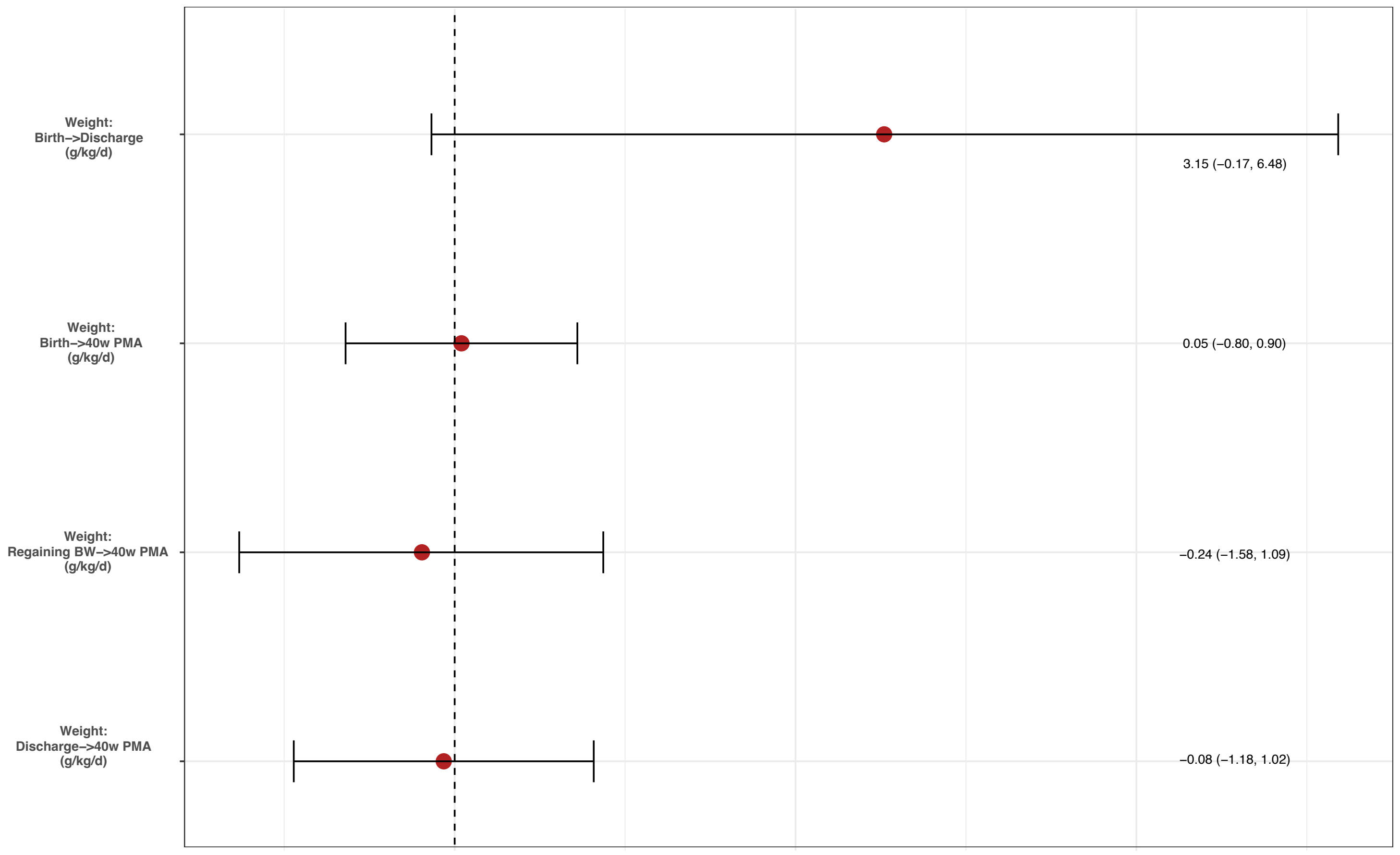

Supplement Figure S2: Adjusted Intention-to-Treat Analyses of Secondary and Safety Outcomes Between Plant-Derived and Bovine-Derived HMF Groups

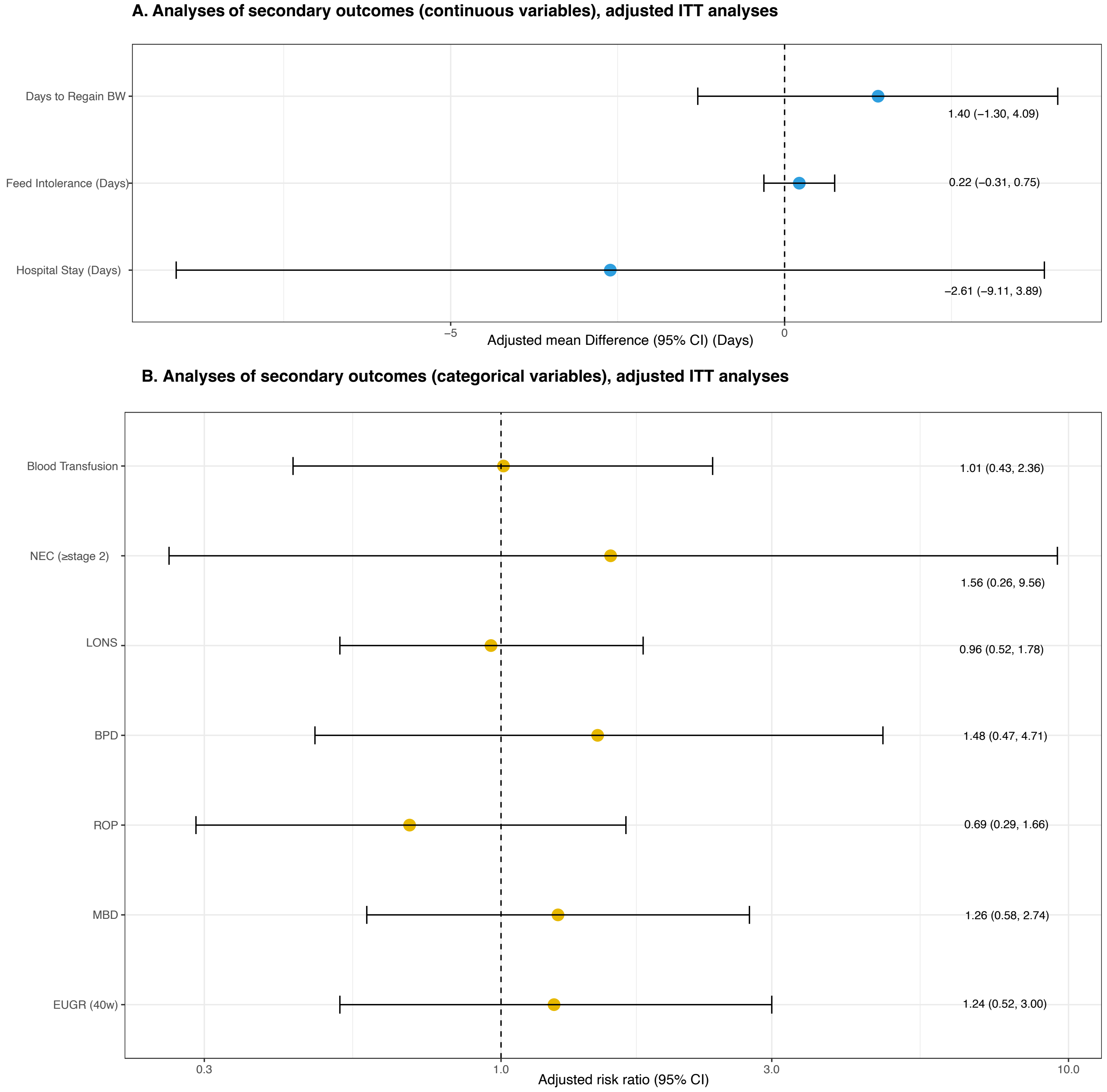

Supplement Figure S3: Adjusted Intention-to-Treat Analysis for Time to Regain Birth Weight (Kaplan-Meier Survival Estimates) Between Plant-Derived and Bovine-Derived HMF Groups

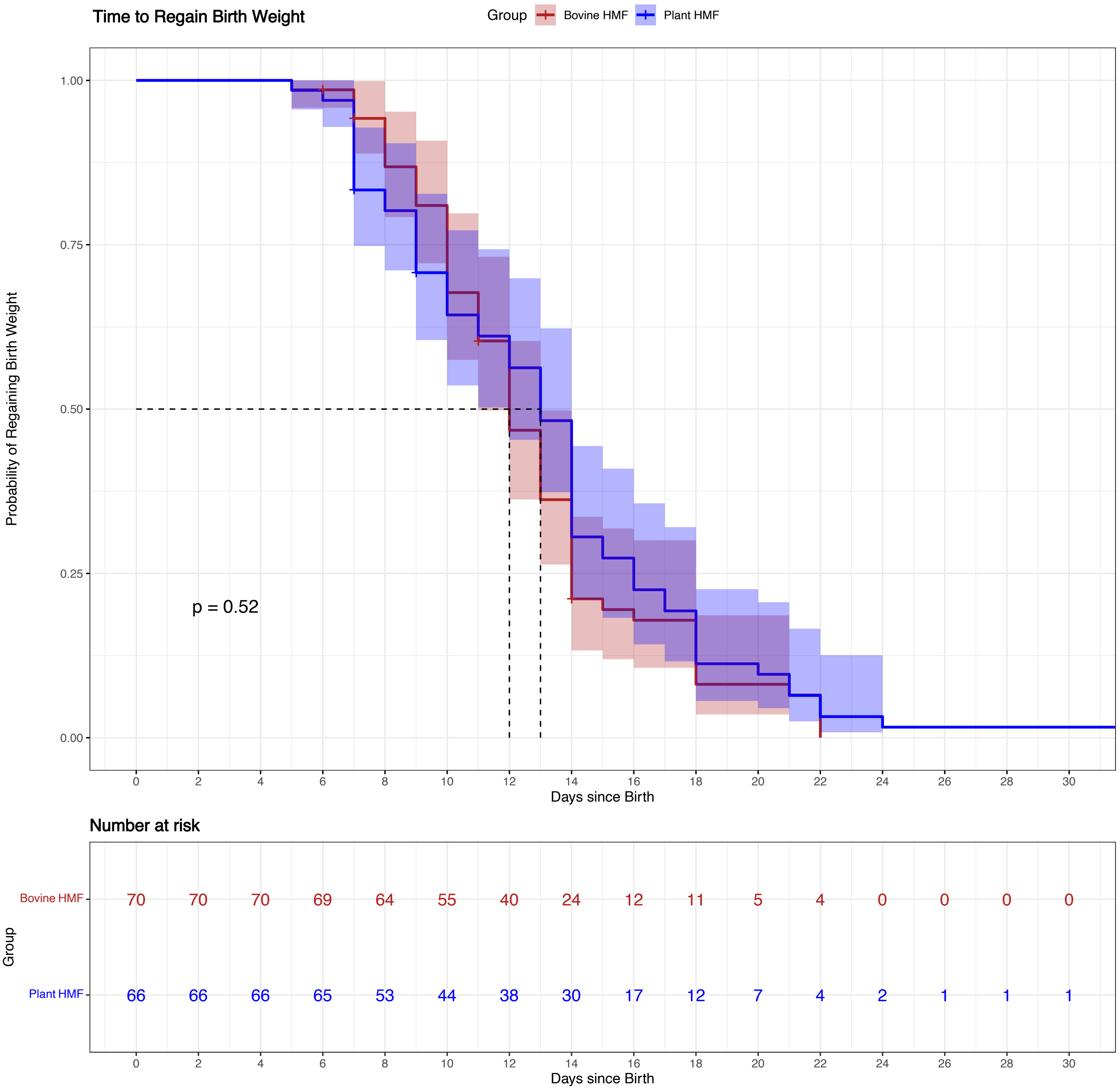

Supplement Figure S4: Adjusted Intention-to-Treat Subgroup Analyses of Growth Velocities (Weight, Length, and Head Circumference) Between Plant-Derived and Bovine-Derived HMF Groups

Subgroup Analyses of anthropometric variables (ITT- Adjusted analyses)

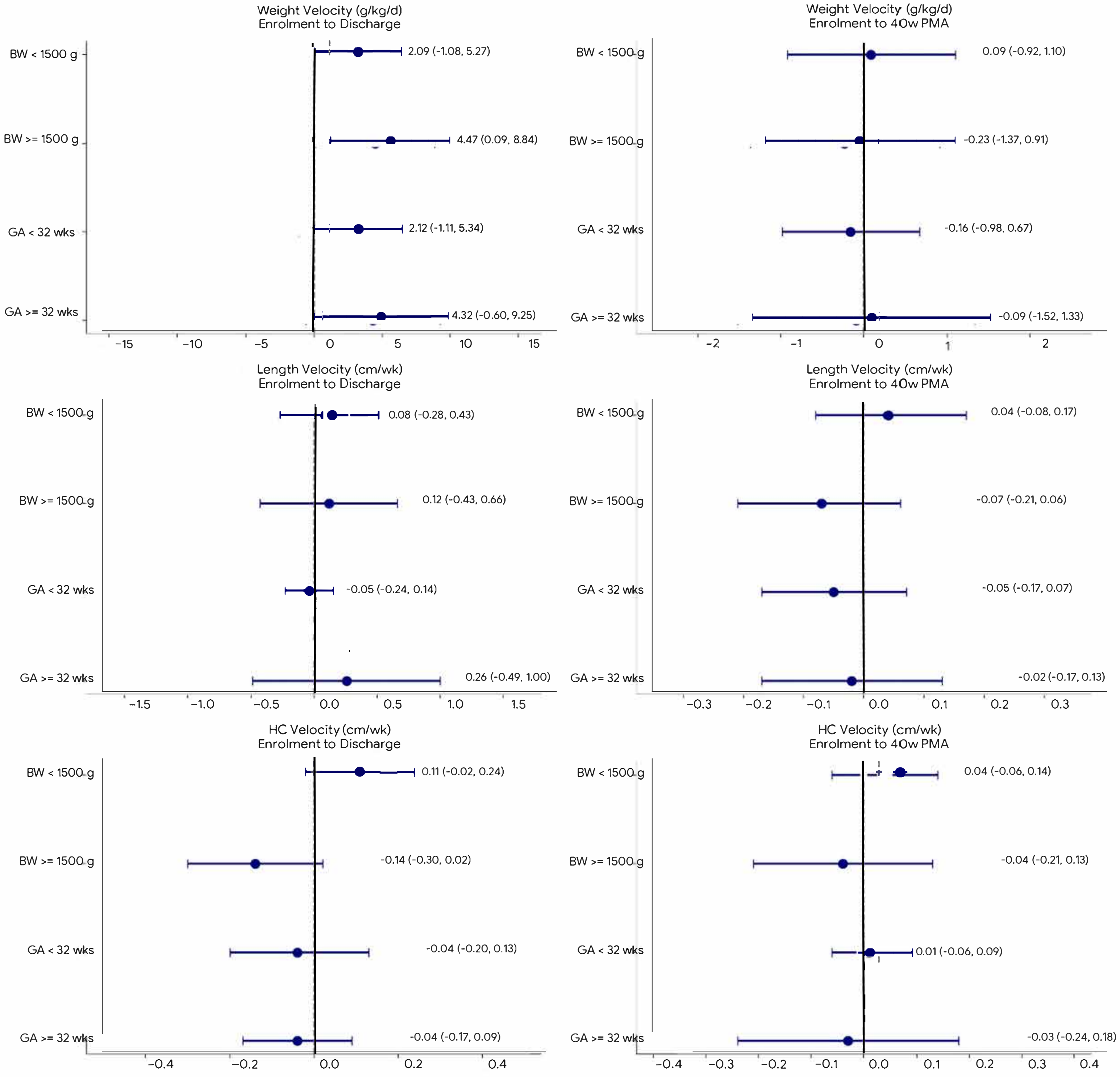

Supplement Figure S5: Adjusted and Unadjusted Per-Protocol Analyses of Growth Velocities (Weight, Length, and Head Circumference) Between Plant-Derived and Bovine-Derived HMF Groups

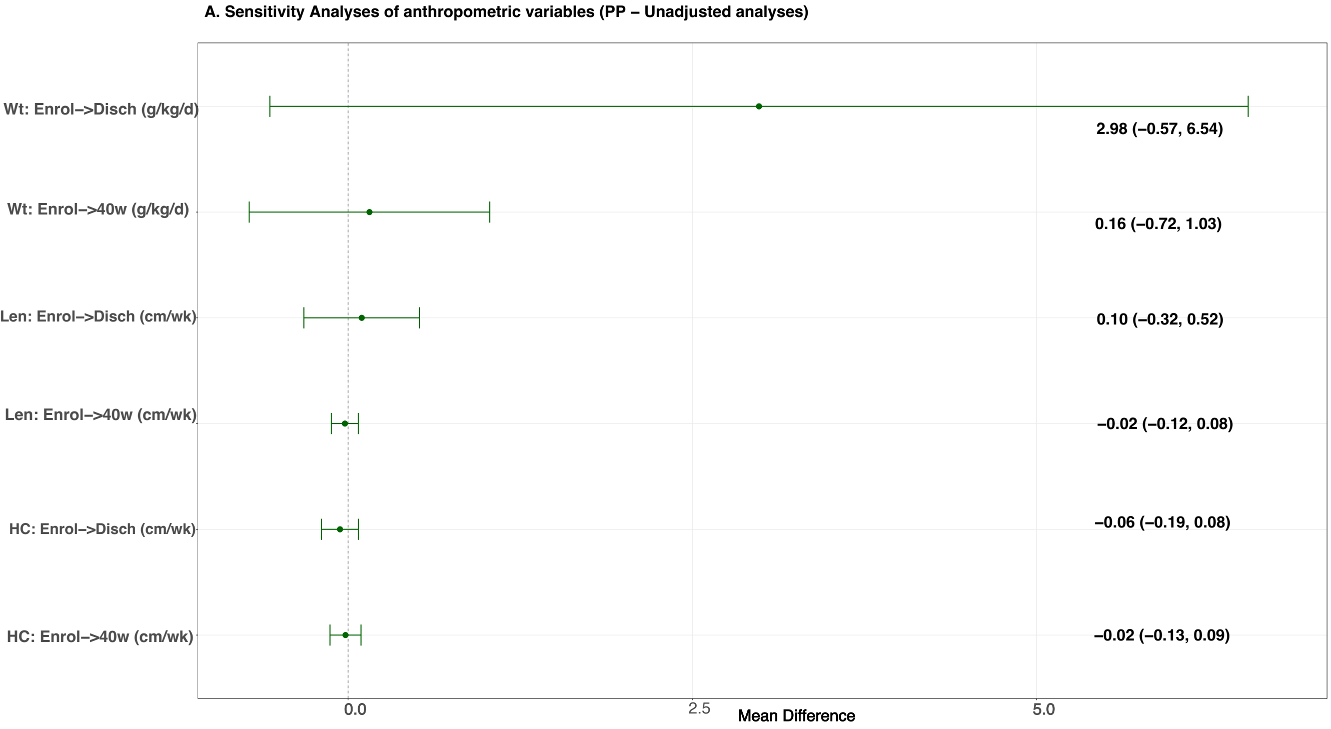

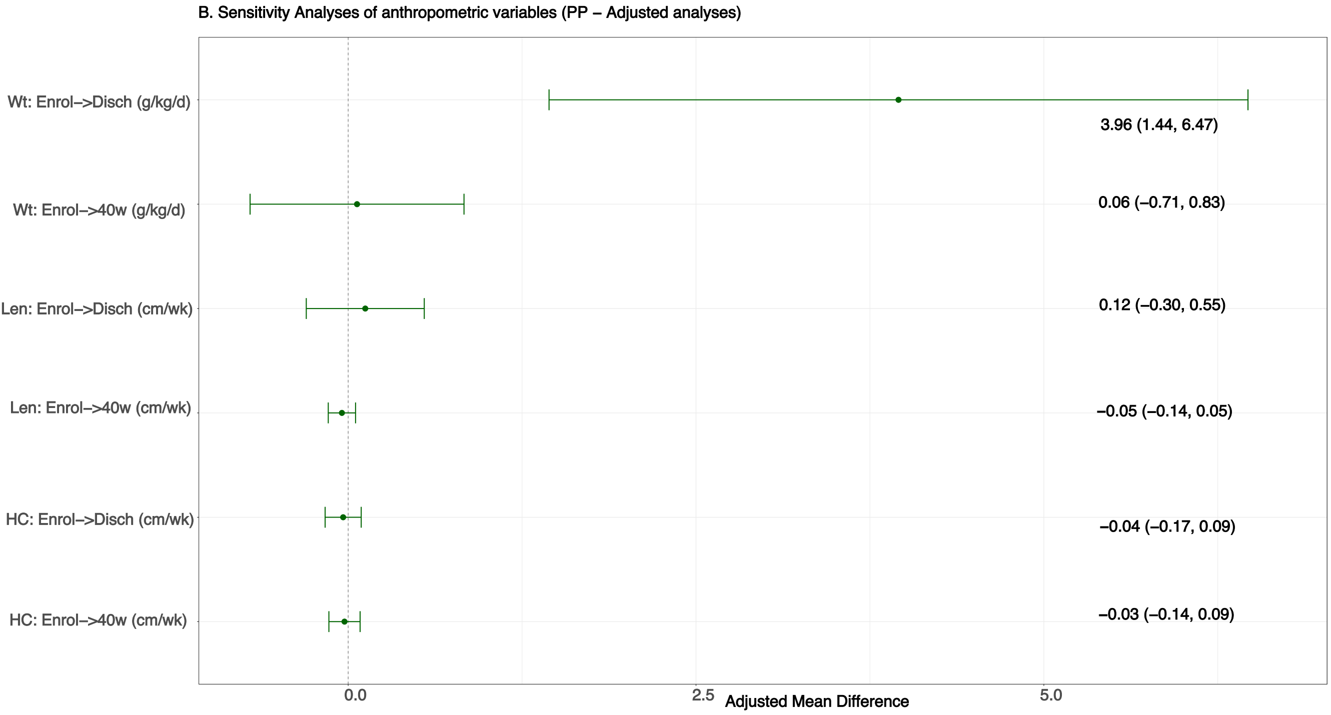

Supplement Figure S6: Adjusted Per-Protocol Subgroup Analyses of Growth Velocities (Weight, Length, and Head Circumference) Between Plant-Derived and Bovine-Derived HMF Groups

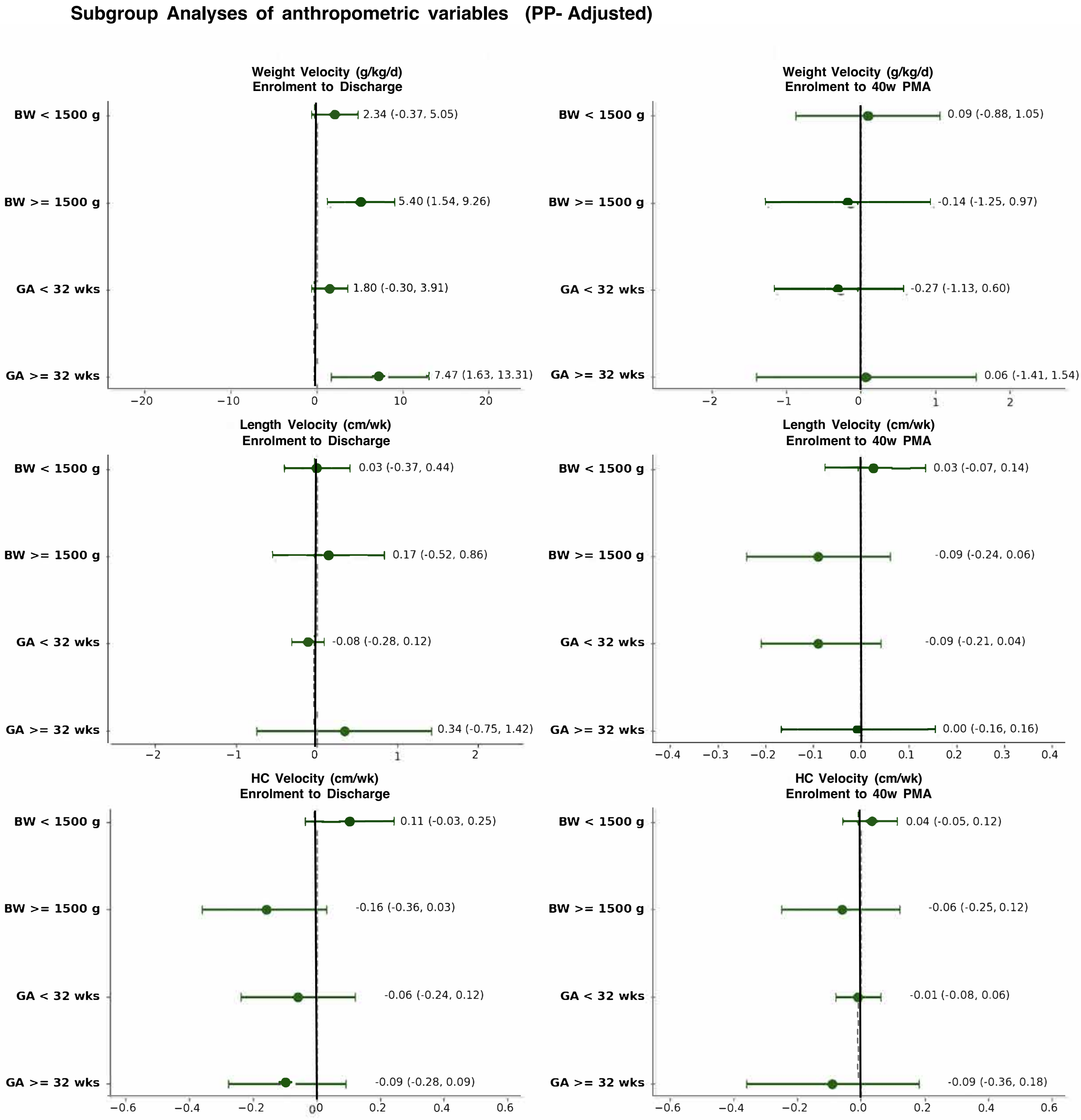

Supplement Figure S7: Unadjusted Longitudinal Growth Trajectories Between Plant-Derived vs. Bovine-Derived HMF groups.

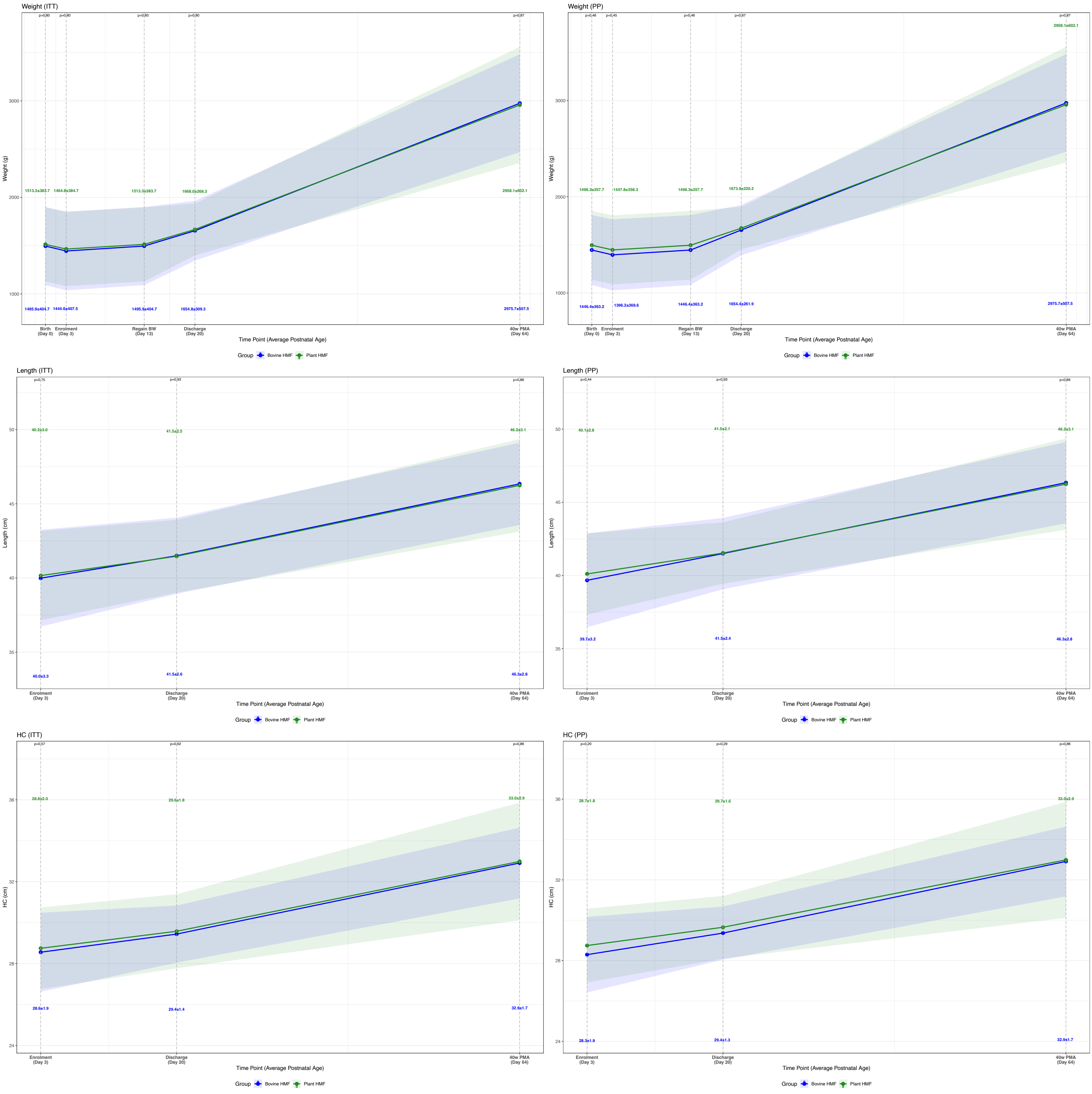

Supplement Table S1: Adjusted Intention-to-Treat Subgroup Analyses of Growth Velocities (Weight, Length, and Head Circumference) Between Plant-Derived and Bovine-Derived HMF Groups

Anthropometric parameter	Subgroup	aMD (95% CI)<br>(Plant HMF (n=66) vs. Bovine HMF (n=70))	p-value
**Weight gain (g/kg/d): Enrolment until discharge**	Overall	**3.20 (0.46, 5.95)**	**0.02**
	BW < 1500 g	2.09 (-1.08, 5.27)	0.20
	BW ≥ 1500 g	**4.47 (0.09, 8.84)**	**0.04**
	GA < 32 w	2.12 (-1.11, 5.34)	0.20
	GA ≥ 32 w	4.32 (-0.60, 9.25)	0.08
**Weight gain (g/kg/d): Enrolment until 40 weeks' PMA**	Overall	-0.03 (-0.81, 0.75)	0.94
	BW < 1500 g	0.09 (-0.92, 1.10)	0.86
	BW ≥ 1500 g	-0.23 (-1.37, 0.91)	0.69
	GA < 32 w	-0.16 (-0.98, 0.67)	0.71
	GA ≥ 32 w	-0.09 (-1.52, 1.33)	0.90
**Length increment (cm/w): Enrolment until discharge**	Overall	0.11 (-0.24, 0.45)	0.54
	BW < 1500 g	0.08 (-0.28, 0.43)	0.67
	BW ≥ 1500 g	0.12 (-0.43, 0.66)	0.67
	GA < 32 w	-0.05 (-0.24, 0.14)	0.58
	GA ≥ 32 w	0.26 (-0.49, 1.00)	0.50
**Length increment (cm/w): Enrolment until 40 weeks' PMA**	Overall	-0.03 (-0.12, 0.07)	0.59
	BW < 1500 g	0.04 (-0.08, 0.17)	0.51
	BW ≥ 1500 g	-0.07 (-0.21, 0.06)	0.29
	GA < 32 w	-0.05 (-0.17, 0.07)	0.43
	GA ≥ 32 w	-0.02 (-0.17, 0.13)	0.79
**HC increment (cm/w): Enrolment until discharge**	Overall	-0.02 (-0.13, 0.09)	0.71
	BW < 1500 g	0.11 (-0.02, 0.24)	0.10
	BW ≥ 1500 g	-0.14 (-0.30, 0.02)	0.09
	GA < 32 w	-0.04 (-0.20, 0.13)	0.66
	GA ≥ 32 w	-0.04 (-0.17, 0.09)	0.50
**HC increment (cm/w): Enrolment until 40 weeks' PMA**	Overall	-0.00 (-0.10, 0.10)	0.99
	BW < 1500 g	0.04 (-0.06, 0.14)	0.41
	BW ≥ 1500 g	-0.04 (-0.21, 0.13)	0.64
	GA < 32 w	0.01 (-0.06, 0.09)	0.72
	GA ≥ 32 w	-0.03 (-0.24, 0.18)	0.78

Supplement Table S2: Adjusted and Unadjusted Per-Protocol Analyses of Growth Velocities (Weight, Length, and Head Circumference) and Weight Gain Velocity Over Time Between Plant-Derived and Bovine-Derived HMF Groups

**Primary outcomes**			
**Anthropometric parameter**	**Time period**	**Unadjusted MD (95% CI) (Plant HMF (n=55) vs. Bovine HMF (n=58))**	**p-value**
**Weight gain (g/kg/d)**	Enrolment until discharge	2.98 (-0.57, 6.54)	0.10
	Enrolment until 40 weeks' PMA	0.16 (-0.72, 1.03)	0.72
**Length increment (cm/w)**	Enrolment until discharge	0.10 (-0.32, 0.52)	0.64
	Enrolment until 40 weeks' PMA	-0.02 (-0.12, 0.08)	0.64
**HC increment (cm/w)**	Enrolment until discharge	-0.06 (-0.19, 0.08)	0.39
	Enrolment until 40 weeks' PMA	-0.02 (-0.13, 0.09)	0.74
**Anthropometric parameter**	**Time period**	**aMD (95% CI) (Plant HMF (n=55) vs. Bovine HMF (n=58))**	**p-value**
**Weight gain (g/kg/d)**	Enrolment until discharge	**3.96 (1.44, 6.47)**	**0.002**
	Enrolment until 40 weeks' PMA	0.06 (-0.71, 0.83)	0.88
**Length increment (cm/w)**	Enrolment until discharge	0.12 (-0.30, 0.55)	0.58
	Enrolment until 40 weeks' PMA	-0.05 (-0.14, 0.05)	0.30
**HC increment (cm/w)**	Enrolment until discharge	-0.04 (-0.17, 0.09)	0.55
	Enrolment until 40 weeks' PMA	-0.03 (-0.14, 0.09)	0.61
**Secondary weight outcomes**	**Time period**	**aMD (95% CI) (Plant HMF (n=55) vs. Bovine HMF (n=58))**	**p-value**
**Weight gain (g/kg/d)**	Birth until discharge	1.81 (-1.10, 5.21)	0.24
	Birth until 40w PMA	-0.16 (-0.98, 0.69)	0.75
	Day of regaining BW until 40w PMA	0.09 (-0.94, 1.15)	0.86
	Discharge until 40w PMA	-0.12 (-1.18, 0.99)	0.82

Supplement Table S3: Adjusted Per-Protocol Subgroup Analyses of Growth Velocities (Weight, Length, and Head Circumference) and Weight Gain Velocity Over Time Between Plant-Derived and Bovine-Derived HMF Groups

Anthropometric parameter	Subgroup	aMD (95% CI) (Plant HMF (n=55) vs. Bovine HMF (n=58))	p-value
**Weight gain (g/kg/d): Enrolment until discharge**	Overall	**3.96 (1.47, 6.44)**	**0.002**
	BW < 1500 g	2.34 (-0.37, 5.05)	0.09
	BW ≥ 1500 g	**5.40 (1.54, 9.26)**	**0.006**
	GA < 32 w	1.80 (-0.30, 3.91)	0.09
	GA ≥ 32 w	**7.47 (1.63, 13.31)**	**0.01**
**Weight gain (g/kg/d): Enrolment until 40 weeks' PMA**	Overall	0.02 (-0.73, 0.77)	0.95
	BW < 1500 g	0.09 (-0.88, 1.05)	0.86
	BW ≥ 1500 g	-0.14 (-1.25, 0.97)	0.80
	GA < 32 w	-0.27 (-1.13, 0.60)	0.55
	GA ≥ 32 w	0.06 (-1.41, 1.54)	0.93
**Length increment (cm/w): Enrolment until discharge**	Overall	0.12 (-0.30, 0.54)	0.57
	BW < 1500 g	0.03 (-0.37, 0.44)	0.87
	BW ≥ 1500 g	0.17 (-0.52, 0.86)	0.63
	GA < 32 w	-0.08 (-0.28, 0.12)	0.42
	GA ≥ 32 w	0.34 (-0.75, 1.42)	0.54
**Length increment (cm/w): Enrolment until 40 weeks' PMA**	Overall	-0.04 (-0.13, 0.06)	0.44
	BW < 1500 g	0.03 (-0.07, 0.14)	0.53
	BW ≥ 1500 g	-0.09 (-0.24, 0.06)	0.24
	GA < 32 w	-0.09 (-0.21, 0.04)	0.16
	GA ≥ 32 w	0.00 (-0.16, 0.16)	1.00
**HC increment (cm/w): Enrolment until discharge**	Overall	-0.04 (-0.16, 0.09)	0.58
	BW < 1500 g	0.11 (-0.03, 0.25)	0.14
	BW ≥ 1500 g	-0.16 (-0.36, 0.03)	0.10
	GA < 32 w	-0.06 (-0.24, 0.12)	0.48
	GA ≥ 32 w	-0.09 (-0.28, 0.09)	0.31
**HC increment (cm/w): Enrolment until 40 weeks' PMA**	Overall	-0.02 (-0.13, 0.09)	0.67
	BW < 1500 g	0.04 (-0.05, 0.12)	0.41
	BW ≥ 1500 g	-0.06 (-0.25, 0.12)	0.50
	GA < 32 w	-0.01 (-0.08, 0.06)	0.77
	GA ≥ 32 w	-0.09 (-0.36, 0.18)	0.51
